# Actions of sesquiterpene lactones isolated from *Moquiniastrum polymorphum* subsp. *floccosum* in MCF7 cell line and their potentiating action on doxorubicin

**DOI:** 10.1186/s40360-017-0156-3

**Published:** 2017-06-29

**Authors:** Mariana de Oliveira Mauro, Renata Matuo, Natan de David, Regiane Lauriano Batista Strapasson, Rodrigo Juliano Oliveira, Maria Élida Alves Stefanello, Cândida Aparecida Leite Kassuya, Maria de Fátima de Cepa Matos, Fábio José Carvalho Faria, Deiler Sampaio Costa

**Affiliations:** 1 Programa de Pós-Graduação em Biotecnologia e Biodiversidade, Doutorado Rede Pró Centro-Oeste, Universidade Federal de Mato Grosso do Sul (UFMS), Campo Grande, Mato Grosso do Sul 79070-900 Brazil; 2 Programa de Mestrado em Farmácia, Centro de Ciências Biológicas e da Saúde (CCBS), Universidade Federal de Mato Grosso do Sul (UFMS), Campo Grande, Mato Grosso do Sul Brazil; 3 Departamento de Química, Universidade Federal do Paraná (UFPR), Curitiba, Paraná Brazil; 4 Centro de Estudos em Células Tronco, Terapia Celular e Genética Toxicológica (CeTroGen), Hospital Universitário Maria Aparecida Pedrossian (HUMAP), Universidade Federal de Mato Grosso do Sul (UFMS), Campo Grande, Mato Grosso do Sul Brazil; 5 Faculdade de Ciências da Saúde, Universidade Federal da Grande Dourados (UFGD), Dourados, Mato Grosso do Sul Brazil; 6 Faculdade de Medicina Veterinária e Zootecnia (FAMEZ), Universidade Federal de Mato Grosso do Sul (UFMS), Campo Grande, Mato Grosso do Sul Brazil

**Keywords:** Moquiniastrum, Breast cancer, Sesquiterpene lactones, Cytotoxicity, Apoptosis

## Abstract

**Background:**

In order to obtain better clinical results in anticancer therapies, polychemotherapy or combination therapies are used. For this, the combinations are required to increase the efficacy and reduce the adverse reactions of the associated chemotherapies. The aim of this study was to evaluate the cytotoxic, apoptotic and (anti)proliferative potential of two sesquiterpene lactones isolated from *Moquiniastrum polymorphum*, 11,13-diidrozaluzanin C (1) and gochnatiolide C (2), and their associations with chemotherapeutic agents irinotecan, tamoxifen, cisplatin, 5-fluouracyl and doxorubicin in the tumoral lineage of MCF-7 breast adenocarcinoma.

**Methods:**

The analyses were performed by MTT cytotoxicity assays, drug combination index (CI), apoptosis morphological assay and cell proliferation assay. Treatments were evaluated with short exposure times (4 h), followed or not by recovery in drug-free medium for 24 h. For the cell viability assay the statistical analysis was performed using software INSTAT, and the ANOVA/Tukey test was applied. Combination Indices (CI) was made using CompuSyn software and demonstrated through isoboles. The assays that evaluated cell death and proliferation used statistical analysis SAS 9.4 (Statistical Analysis System), and the procedure adopted was PROC NPAR1WAY. The Wilcoxon test at 5% level was applied for comparing statistical differences.

**Results:**

The results demonstrated that the compounds decrease cell viability and increase their action when associated with irinotecan, tamoxifen and doxorubicin (CI < 1 and CI = 1). In periods of 4 h-exposure, the compounds cause cell death by apoptosis and after 24 h, they increase the mean number of cells in programmed cell death, especially when treated with 2. In addition, the association with doxorubicin increases the apoptotic potential induced by tested compounds. Both isolates had effect on the reduction of the number of mitoses, especially when 2 at its highest concentration is associated with doxorubicin.

**Conclusions:**

Finally, these compounds are presented as potential agents in chemotherapy combined with doxorubicin, since they trigger the mechanism of apoptosis, which, through the mechanism of action of sesquiterpene lactones, leads to a reduction in toxicity. In addition, the tested compounds have the ability to exert a synergistic action with doxorubicin, possibly by down-regulating the drug resistance mechanisms.

## Background

Cancer is the largest public health problem in the Americas, and breast cancer is the most common type among women, accounting for about 25% of new cases each year [1, 2].

Therapeutic strategies for this type of neoplasia have posed a challenge because the disease is complex and has several subtypes, which directly influences the choice of therapy, clinical outcomes and prognosis [1, 3]. This facts lead to the search for effective therapies.

In general, chemotherapeutic agents are toxic, and this effect becomes more enhanced in rapidly proliferating tissues as cell cycles are shorter. This phenomenon causes greater toxicity of the agents in tissues such as mucosae and cells of the immunological system since they are capable of accumulating more damage. Another important aspect is the ability of the neoplastic cell to undergo mutations that can trigger resistance to chemotherapeutics, thus reducing therapeutic efficacy [4, 5]. This condition is associated with the fact that cell cycles of tumor cells occur within a shorter period of time and more frequently [5].

In order to obtain better clinical results in anticancer therapies, polychemotherapy or combination therapies are used, which consist of the association of more than one cytostatic/cytotoxic agent with distinct mechanisms of action so as to delay tumor resistance [6] and to better induce tumor cell death or tumor eradication [7]. For this, the combinations are required to increase the efficacy and reduce the adverse reactions of the associated chemotherapies, which generates additive and/or synergistic effects [8].

The plants, widely used in traditional medicine for the treatment of diseases, are important sources of bioactive substances and, among them, secondary metabolites such as sesquiterpene lactones are highlighted for having a recognized antitumor effect [9, 10].

Approximately 1500 genera and 23,000 species constitute the plants of the family *Asteraceae*, the species *Moquiniastrum polymorphum*, native to Brazil, Paraguay, Uruguay and Argentina, is used in folk medicine for the treatment of respiratory diseases, sore throat and gastritis [11] and has anti-inflammatory, antispasmodic and antimicrobial action [12, 13], besides antioxidant, cytotoxic and antitumor activity [11, 14].

Previous studies of our group have evaluated the antiproliferative capacity of six sesquiterpene lactones isolated from *Moquiniastrum polymorphum* [10], among them 11,13-dihydrozaluzanin C (1) and 10-desoxigochnatiolide A (2, gochnatiolídeo C), also used in this study. The assay was performed in 10 different human cancer cell lines, showing that 1 was inactive, while 2 presented an activity similar to that of doxorubicin, which was used as a positive control. A lactones mixture also showed antitumor activity against the Walker-256 tumor in rats [14].

To this end, cytotoxic and cytostatic activities were evaluated, and also the ability to induce apoptosis of sesquiterpene lactones 11,13-diidrozaluzanin C (1) and gochnatiolide C (2) isolated from *Moquiniastrum polymorphum* subsp. *floccosum.* Their effect in association with different commercial antitumor agents was also assessed.

## Methods

### Cell line and culture conditions

MCF-7 breast adenocarcinoma cell line and cell cultures were kindly provided by Professor Maria de Fátima Cepa Matos, of the Laboratory of Molecular Biology of the Center of Biological Sciences of the Federal University of Mato Grosso do Sul. The cells were cultured in Dulbecco’s Modified Eagle Medium (DMEM-Gibco®), supplemented with 10% fetal bovine serum (v/v), 0.1% penicillin (100U/ml)/streptomycin (100 μg/ml) (v/v), in incubator at 37 °C with 5% CO_2_ atmosphere.

### Chemical agents

**Fig. 1:**
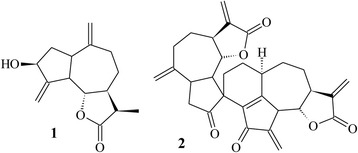
Chemical structures of 11,13-dihydrozaluzanin C (1) and gochnatiolide C (2)

As damage-inducing agents, the following chemotherapeutics were used at their respective concentrations of IC_50_ previously determined by pilot studies on MCF-7 cells: 0.3 μM doxorubicin (Bergamo®); 5.0 μM cisplatin (Gunther®); 5-Fluouracyl 1.25 μM (Biosynthetic®); 12 μM Tamoxifen (Sanofi Aventis®); and 5 μM irinotecan (Janssen Cilag®) (Fig. 1).

The sesquiterpene lactones of *M. polymorphum* were obtained as previously described [10, 15]. Compound 1 (11,13-diidrozaluzanin C) was tested at concentrations of 40; 100; 200; 300 and 400 μM, and compound 2 (gochnatiolide C) at concentrations of 0.5; 1.0; 2.0; 3.0 and 4.0 μM, diluted in dimethyl sulfoxide (DMSO) at 1.0% in all experiments. DMSO was also added in the same percentage to controls. The concentrations tested of compounds 1 and 2 were defined according to previous results [10].

### Cell viability assay

Cell viability was determined by colorimetric test MTT (3-(4,5-dimetilltiazol-2-il)-2,5-difeniltetrazol bromide), based on the protocol of Poindessous et al. [16] and Mauro et al. [17]. 3 × 10^3^ MCF-7 cells were seeded per well in 96-well plates and maintained for 24 h in a CO_2_ incubator for stabilization. The treatments were performed for 72 h at different doses of chemotherapeutic agents, being thus distributed for the determination of IC_50_: doxorubicin (Bergamo®) 0.1; 0.3; 0.4; 0.5 μM; cisplatin (Gunther®) 2.5; 5.0; 10; 25; 50 μM; 5-Fluouracyl (Biosynthetic®) 0.1; 1.0; 2.5; 5; 10 μM; tamoxifen (Sanofi Aventis®) 2.5; 5; 7.5; 10 μM and irinotecan (Janssen Cilag®) 10; 20; 40; 60 μM.

In the same way was carried out for the isolated compounds, using the following concentrations: compound 1 (400; 100; 200; 300 and 400 μM) and 2 (0.5; 1.0; 2.0; 3.0 and 4.0 μM) and simultaneously associated with IC_50_ of different commercial chemotherapeutics (determined by the methodology explained above). At the end of treatments, the plates were incubated with MTT 3.5 mg mL^−1^ for 4 h. The culture medium was removed and DMSO was added to permit absorbance reading in a spectrophotometer (Robonik®) in a 540 nm filter. For each experiment three independent replicates were performed in quintuplicates. Statistical analysis was performed using software INSTAT, and the ANOVA/Tukey test was applied.

From the cell viability curves of 1 and 2 in combination with the different chemotherapeutic agents, the Combination Indices (CI) were calculated from the values of the affected cell fractions (FA), where: CI < 1 indicates synergism; CI = 1 indicates additive effect and CI > 1 indicates antagonistic effect. The calculation dos CI and normalized isobolograms were made according to the method described by Chou and Talalay [18] using CompuSyn software (http://www.combosyn.com/).

### Evaluation of cell death

Cell deaths were evaluated by morphological assay according to the protocol of Oliveira et al. [19], and classified into apoptotic or necrotic ones. The technique of differential staining with acridine orange and ethidium bromide was used for detecting cell viability, apoptosis and necrosis indices. A total of 5 × 10^5^ cells were seeded in 12-well plates and kept in incubator for 24 h. The evaluation occurred in two different times: i) after 4 h of treatment (4 h), and ii) 4 h-treatment followed by 24 h in drug-free medium (4 h + 24 h). For each experiment, two independent replicates were performed, using the doses of 14, 28 and 56 μM for 1; and 0.5; 1.0 and 2.0 μM for 2. These same doses were associated with 0.3 μM doxorubicin. The cells were collected by trypsinization, centrifuged at 1200 rpm for 5 min and the supernatant was discarded. The slides were prepared with 20 μL of cell suspension and 2 μL of dye containing ethidium bromide (100 μg ml^−1^) and acridine orange (100 μg mL^−1^) in a ratio 1:1. Two independent repetitions were performed with two replicates, and 100 cells per replicate were analyzed under an epifluorescence microscope (MOTIC®, Model BA410) in a 400x magnification.

Cell classification was performed according to the following description: (i) living cells with functional membrane have uniform green coloration in their nucleus; (ii) cells in initial apoptosis with functional membrane, but with DNA fragmentation, show a green coloration in nucleus and cytoplasm, with a visible marginalization of their nuclear content; (iii) cells in final apoptosis present orange-stained areas both in the cytoplasm and in the sites where the chromatin is condensed in the nucleus, which distinguishes them from necrotic cells; (iv) necrotic cells have uniform orange staining in the nucleus.

Statistical analysis was performed using SAS 9.4 (Statistical Analysis System), and the procedure adopted was PROC NPAR1WAY. The Wilcoxon test at 5% level was applied for comparing statistical differences.

### Cell Proliferation Assay

For assessing cell proliferation, the cells were labeled with 5-bromo deoxyuridine (BrdU), a synthetic nucleoside analogous to thymidine, incorporated into the newly synthesized DNA strands of cells in replication process as a replacement for thymidine. Thus, the greater the incorporation of BrdU, the greater the proliferative activity of the cells analyzed, since this points to an increased DNA synthesis.

In the assay, 7.5 × 10^5^ cells were seeded on coverslips in 6-well plates and maintained in culture for a period of 24 h for stabilization and fixation of cells on the coverslips. They were then treated with 1 and 2 at concentrations of 28 μM and 1 μM, respectively; and these same concentrations were associated with doxorubicin (0.3 μM) for 6 h. BrdU was added in the last 1 h of treatment. After this time, the cells were washed with phosphate buffer, fixed with 4% paraformaldehyde, and the DNA denatured with 2 M HCl was deposited on the coverslip at room temperature. The cells were incubated with PBS containing 5% fetal bovine serum for 2 h, followed by incubation with anti-BrdU antibody for 1 h. The cells were washed with phosphate buffer and incubated with secondary antibody AlexaFluor 568 for 1 h.

For the analysis, 200 cells were counted and separated as BrdU-labeled cells and unlabeled cells. We also calculated the Mitotic Activity Reduction Ratio (MARR) obtained from the average of the control mean divided by the results obtained in the means of tests. Software SAS 9.4 (Statistical Analysis System) was used for statistical analysis; the procedure adopted was PROC NPAR1WAY; and the Wilkoxon test was applied at 5% level to compare statistical differences. The Kruskal-Wallis test was employed for analyzing the statistical significance of the results in both protocols.

## Results

### Determination of the IC_50_ of the commercial chemotherapeutic agents, for association with the isolated compounds

**Fig. 2:**
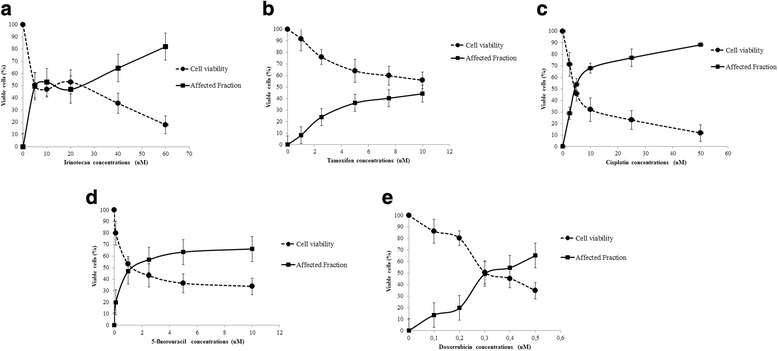
Determination of the IC_50_ of commercial chemotherapeutics in culture of MCF7 cells. Different concentrations of **a** doxorubicin (0.1; 0.3; 0.4; 0.5 μM); **b** cisplatin (2.5; 5.0; 10; 25; 50 μM); **c** 5-Fluouracyl (0.1; 1.0; 2.5; 5; 10 μM); **d** tamoxifen (2.5; 5; 7.5; 10 μM) and **e** irinotecan (10; 20; 40; 60 μM) were tested. Cell viability and affected fraction plots represent the mean ± standard deviation of three independent replicates with five replicates

Figure 2 shows the graphs that indicate the values of affected cells fraction (FA) and viable cells after exposure of cells MCF-7 to different concentrations of commercial chemotherapeutic agents. The point of intersection of these two parameters determines the concentration of IC_50_, in order to determine this concentration is necessary for the values of FA and of viable cells should be 50%, converging at a common point of the chart. The IC_50_ of chemotherapeutic agents were trade thus defined: 0.3 μM doxorubicin; 5.0 μM cisplatin; 5-Fluouracyl 1.25 μM; 12 μM Tamoxifen; and 5 μM irinotecan.

### *M. polymorphum* subsp. *floccosum* isolates decrease the cell viability of the MCF-7 lineage and increase the activity of the chemotherapeutic agents irinotecan, tamoxifen and doxorubicin

**Fig. 3:**
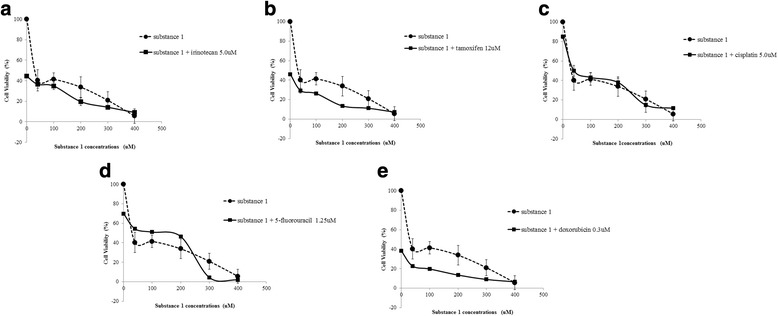
Effects of 1 on the cell viability of the MCF7 cell line. Different concentrations of 1 (40; 100; 200; 300 and 400 μM) were combined with the IC 50 of the chemotherapeutic agents **a** irinotecan, **b** tamoxifen, **c** cisplatin, **d** 5-Fluouracyl and **e** doxorubicin. Cell viability plots represent the mean ± standard deviation of three independent replicates with five replicates

**Fig. 4:**
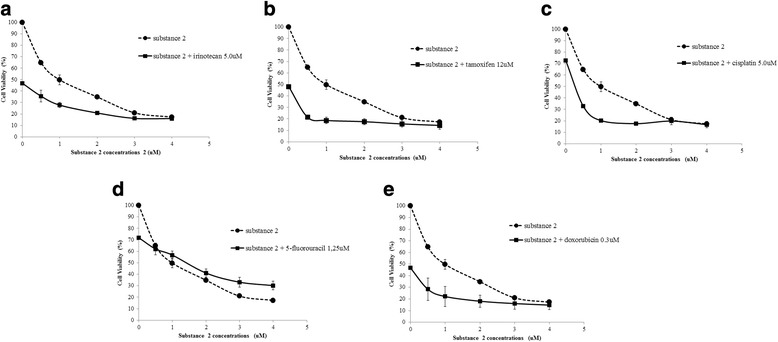
Effects of 2 on the cell viability of the MCF7 cell line. Different concentrations of 2 (0.5; 2; 3 and 4 μM) were combined with the IC_50_ of the chemotherapeutic agents **a** irinotecan, **b** tamoxifen, **c** cisplatin, **d** 5-Fluouracyl and **e** doxorubicin. Cell viability plots represent the mean ± standard deviation of three independent replicates with five replicates

The results of the test of MTT assay performed with the compounds 1 and 2 can be viewed on the panels formed by the graphics of cellular viability available in Fig. 3 (lactone 1) and Fig. 4 (lactone 2). The MTT assay demonstrated that the sesquiterpene lactone 1 (Fig. 3) reduced the cell viability of MCF-7 to less than 50% the number of cells at the lowest concentration tested (40 μM) after 72 h of treatment, yielding a cell viability value of 40.30%.

In turn, sesquiterpene lactone 2 (Fig. 4) led to a reduction in MCF-7 cell viability in near 50% at concentration of 1 μM (49.78%) after 72 h of exposure to 2.

Supported by the cell viability of each one of the concentrations, IC_50_ was calculated for these two compounds, estimated at 28 μM and 1 μM, respectively.

Compound 1 at 40 μM concentration, in combination with irinotecan (Fig. 3a), tamoxifen (Fig. 3b) and doxorubicin (Fig. 3e), increased the induction of cell death by 3.68%, 11.03% and 17.71%, respectively, when compared with the treatment in which only 1 was added. The association with cisplatin (Fig. 3c) did not alter cell viability. The association with 5-fluorouracyl reduced the ability to induce cell death by 9.75% (Fig. 3d).

In the associated treatments of 2 with the different antineoplastic agents, a reduction of viable cells was observed, especially in the estimated IC_50_ (28 μM) in combinations with irinotecan (Fig. 4a), tamoxifen (Fig. 4b), cisplatin (Fig. 4c), and doxorubicin (Fig. 4e). The increase in non-viable cells, when compared with the compound alone, was 29.41%, 28.37%, 29.6% and 27.61%, respectively. Only the combination with 5-fluorouracyl (Fig. 4d) did not present a decrease in viable cells.

**Fig. 5:**
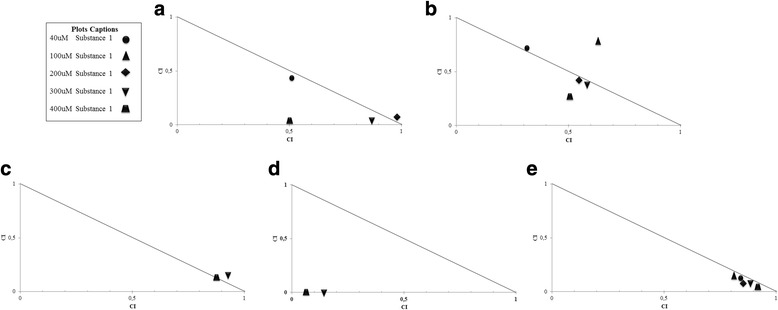
Effects of 1 on the Combination Index (CI) of drugs in MCF7 cell line. Compound 1 concentrations (40; 100; 200; 300 and 400 μM) were combined with the IC_50_ of the chemotherapeutic agents irinotecan **a**, tamoxifen **b**, cisplatin **c**, 5-Fluouracyl **d** and doxorubicin **e**. CI and the normalized isobolograms was calculated using the CompuSyn software, where: CI <1 synergism; CI = 1 additive effect; CI > 1 antagonistic effect

Combinations of 1 with irinotecan and tamoxifen showed additive effect and synergism (Fig. 5a and b) while the association with doxorubicin (Fig. 5e) showed synergism at all concentrations. On the other hand, the combinations of 1 with cisplatin (Fig. 5c) and 5-fluorouracyl (Fig. 5d) presented antagonism and additive effect.

**Fig. 6:**
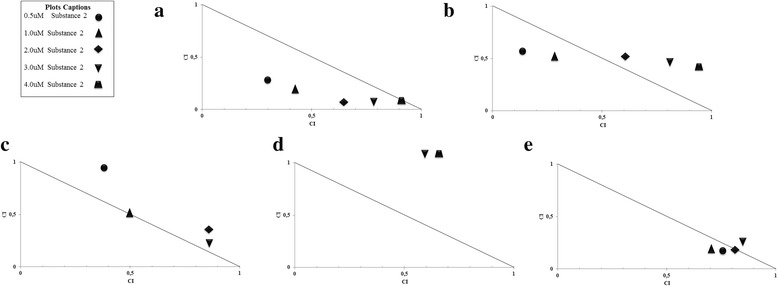
Effects of 2 on drug Combination Index (CI) of MCF7 cell line. The concentrations of 2 (0.5; 2; 3 and 4 μM) were combined with the IC 50 of the chemotherapeutic agents irinotecan **a**, tamoxifen **b**, cisplatin 5-Fluouracyl **d** and doxorubicin **e**. CI and the normalized isobolograms was calculated using the CompuSyn software, where: CI <1 synergism; CI = 1 additive effect; CI > 1 antagonistic effect

Combinations of 2 with irinotecan showed synergism (Fig. 6a), whereas associations with tamoxifen (Fig. 6b) and doxorubicin (Fig. 6e) indicated synergism and additive effect. The combinations of 2 with cisplatin (Fig. 6c) showed additive effect and antagonism, whereas combinations with 5-fluorouracyl showed severe antagonism (Fig. 6d).

### Compounds 1 and 2 induce cell death by apoptosis

The results showed that both sesquiterpene lactones induced cell death by apoptosis after 4 h treatment and in the protocol of 4 h followed by post-incubation in drug-free medium for 24 h (4 + 24 h).

In the treatments with the isolated compounds, carried out with post-incubation, an increase in the number of apoptotic cells was observed. This is especially true at concentrations of 28 μM for 1 and 2.0 μM for 2, reaching values of 33.5 ± 3.77 and 60.25 ± 10.03 cells in apoptosis, respectively. Although both isolates induce apoptosis, 2 showed even higher rates of cell death compared with 1 (Table 1).

**Table 1:** Mean and standard deviation of apoptotic MCF-7 cells after 4 and 24 h exposure at three concentrations of compound 1 (11,13-diidrozaluzanin C) and 2 (Gochnatiolide C) associated with doxorubicin

Concentrations	Apoptosis Assay		Comparison between 4 and 24 h
	Mean of apoptotic cells ± SD (4 h)	Mean of apoptotic cells ± SD (24 h)	
Control	4.00 ± 1.87^a^	10.75 ± 2.86^a^	*p* > 0,05
Doxorrubicin (D) - 0.3 μM	33.25 ± 5.34^b^	61.75 ± 10.82^b^	*
11,13-diidrozaluzanin C (Compound 1)			
Compound 1 – 14 μM	16.00 ± 1.87^c^	23.25 ± 3.83^b^	*
Compound 1–28 μM	24.25 ± 5.71^b,c^	33.5 ± 3.77^c^	*p* > 0,05
Compound 1–56 μM	21.5 ± 4.83^b,c^	17.5 ± 2.95^a^	*p* > 0,05
Compound 1–14 μM + D 0.3 μM	47.00 ± 6.07^b^	47.25 ± 5.21^b^	*p* > 0,05
Compound1 - 28 μM+ D 0.3 μM	39.75 ± 8.19^b^	51.25 ± 10.2^b^	*p* > 0,05
Compound1 - 56 μM + D 0.3 μM	52.25 ± 9.8^b^	31.5 ± 4.6^c^	*
Gochnatiolide C (Compound 2)			
Compound 2–0.5 μM	24.50 ± 2.69^b^	40.00 ± 4.94^c^	*
Compound 2–1.0 μM	41.75 ± 10.05^b,c^	51.50 ± 7.69^b^	*p* > 0,05
Compound 2–2.0 μM	53.25 ± 6.13^c^	60.25 ± 10.03^b^	*p* > 0,05
Compound 2–0.5 μM + D 0.3 M	31.25 ± 4.20^b^	31.25 ± 5.76^c^	*p* > 0,05
Compound 2–1.0 μM + D 0.3 μM	40.00 ± 5.43^b^	45.25 ± 4.14^c^	*p* > 0,05
Compound 2–2.0 μM + D 0.3 μM	46.25 ± 4.44^b,c^	62.00 ± 7.51^b^	*

Frequency of apoptotic cells after 4 and 24 h of test - free medium. SD: Standard Deviation. Compound 1 at concentrations of 14; 28 and 56 μM Compound 2 at concentrations of 0.5; 1.0 and 2.0 μM combined or not with doxorubicin IC 50 in MCF7 breast cancer cell line. We analyzed 100 cells, the data represent mean and standard deviation of the average of two independent replicates with two replicates. Different letters indicate statistically significant differences. The results were evaluated by NPAR1WAY/Wilkoxon. Significance * *p* <0.05

In the combined treatments, 1 was seen to increase the amount of cells in apoptosis compared with doxorubicin after 4 h of treatment. However, the same was not seen for all post-incubation treatments, only for the association of 28 μM 1 with doxorubicin.

In the combined treatments of 2 and doxorubicin, it was observed a statistically significant increase of cells at programmed cell death at concentration of 2.0 μM in both treatments: 4 h and 4 h plus 24 h in drug-free medium.

When comparing the different protocols (4 h and 4 h + 24 h) as to the mean and standard deviation obtained, a statistically significant reduction (more than half) was detected in the number of cells in apoptosis in the combined treatment of 1 56 μM and doxorubicin. Also statistically significant is the increased apoptotic activity present in the cells treated with doxorubicin and 2.0 μM 2 combined (Table 1).

### The lactones of *M. polymorphum* subsp. *floccosum* decrease cell proliferation

The results of the cell proliferation evaluation trial demonstrated that both 1 and 2 decreased mitotic activity of MCF-7 cells, and the effect of 2 being more pronounced than that of 1, both in isolation and in combination with doxorubicin.

It is observed that the mean cell division is 1.63-fold reduced when 1 is tested alone, and 1.82-fold when tested in combination with doxorubicin. This reduction is 2.08-fold when 2 is tested alone and 2.71-fold when combined with chemotherapy (Table 2).

**Table 2:** Effect of treatment with compound 1 (11,13-diidrozaluzanin C) and 2 (Gochnatiolide C) on the cell cycle in MCF-7 cells associated or not to Doxorubicin. Mean ± standard deviation and reduction ratio of mitotic activity of tested cells

Concentrations	Frequency of cells in mitotic activity	MARR
	Mean ± SD	
Control	77.5 ± 4.03^a^	-
Doxorrubicin (D) - 0.3 μM	35.25 ± 4.5^b^	2.19
11,13-diidrozaluzanin C (Compound 1)		
Compound 1–28 μM	47.5 ± 5.25^c^	1.63
Compound 1–28 μM+ D 0.3 μM	42.5 ± 7.85^b,c^	1.82
Gochnatiolide C (Compound 2)		
Compound 2–1.0 μM	37.25 ± 3.09^b^	2.08
Compound 2–1.0 μM + D 0.3 μM	28.5 ± 7.5^b^	2.71

Frequency of cells in mitotic activity. Compound 1 at the concentration of 28 μM and compound 2 at the concentration of 1.0 μM combined or not with IC 50 of doxorubicin in MCF7 breast adenocarcinoma cells. SD: Standard Deviation. MARR - Mitotic Activity Reduction Ratio was obtained by calculating the ratio from the value of the control mean divided by the results obtained in the means of the tests. We analyzed 100 cells, the table represents mean and standard deviation of the mean of two independent replicates with two replicates. Different letters indicate statistically significant differences. The results were evaluated by NPAR1WAY/Wilkoxon. Significance * *p* <0.05

## Discussion

Breast cancer is a disease whose incidence and mortality have increased in recent years, a fact that burdens the government funds since the treatments are long-lasting and expensive [2]. Chemotherapy with cytotoxic/cytostatic agents may be inefficient. Thus, the search for more effective treatments has become one of the important targets in the study of pharmacogenetics and pharmacogenomics in cancer [4, 5].

Combined therapy is the most frequently used form of anti-cancer treatment to achieve (i) synergistic therapeutic effects; (ii) dose and toxicity reduction; and (iii) delay and/or minimization of drug resistance induction [20, 21]. To that end, sesquiterpene lactones (1 and 2) were tested with commercial chemotherapeutic agents that have different mechanisms of action in order to potentiate their antitumor effects in MCF-7 cells, an *in vitro* screening model for the treatment of breast cancer.

The results demonstrate that, when tested alone, sesquiterpene lactones 1 and 2 reduce cell viability by the MTT assay, being 2 the most active compound. The cytotoxic activity of these compounds have been ever described in previous study using the sulphorhodamine B method [10]. However this study demonstrates in a pioneering way that these two lactones cause cell death by apoptosis (differential assay of cell death − apoptosis x necrosis, differential staining by ethidium bromide and acridine orange) and reduce cell proliferation (cell proliferation assay with BrdU), although with different powers. These findings allow us to infer that the compounds have cytotoxic and cytostatic action in breast tumor cells, which suggests relevant chemotherapeutic action for the treatment of the disease.

In previous studies [22], it has been shown that cytostatic/cytotoxic action of sesquiterpene lactones can be carried out by means of a selective allocation, via the addition of Michel, of the sulfhydryl groups of the lysosomal enzymes. This fact resulted in the reduction of DNA synthesis and consequent decrease in the uncontrolled growth of tumor cells. However, such a mechanism is present in compounds having a double bond conjugated with a carbonyl, which is a structure present only in compound 2.

Because 1 has a different chemical structure, lacking the α-methylene-γ-lactone group, it is understood that its action mechanism is different from that of 2. Previous studies [13] indicate that 1 has anti-inflammatory action determined by the reduction of cytokine TNF-α levels. It is known that this cytokine is determinant in the tumor microenvironment, promoting proliferation and invasion of tumor cells, TNF-α also positively regulates anti-apoptotic proteins. Thus, its reduction by the presence of 1, can lead to the release of cytochrome c, consequently increase of caspase proteins, and finally triggering the apoptotic event [23].

These important antitumor results evidenced for the sesquiterpene lactones (1 and 2) have aroused interest in knowing their effects in association with commercial chemotherapeutic agents, which are cell damage-inducing agents widely used in anticancer therapies.

In the combined studies of 1, this compound was observed to increase the effects of drugs irinotecan, tamoxifen and doxorubicin. From the cell viability curves of these associations, the CIs were calculated, which confirmed the responses obtained by the MTT assay demonstrating additive effect and synergism for these combinations. However the combination of 1 with cisplatin and 5-fluorouracyl has been shown to be antagonistic to these chemotherapeutics. Thus the data suggest that 1 may be an important compound for the development of antitumor therapies combined with irinotecan, tamoxifen and doxorubicin, but our findings discourage its association with cisplatin and 5-fluorouracyl.

In turn, experiments combined with 2 demonstrated potentiation of the effects with irinotecan, tamoxifen, cisplatin and doxorubicin. CI values confirmed the responses obtained in the MTT assay and indicated additive effects for the different concentrations of 1 combined with tamoxifen and cisplatin, and synergism for irinotecan and doxorubicin. The combination with 5-fluorouracyl, however, demonstrated antagonism. Therefore the results discourage the combined use of 2 and 5-fluorouracyl in the development of anticancer therapies.

The variations in the responses of associations (additive effect, synergism or antagonism actions) may occur because of the different mechanisms of action of the chemotherapeutic agents tested and also because of the influence of 1 and 2 on them. Irinotecan belongs to a class of anticancer agents that act directly on enzyme topoisomerase I, causing genomic instability that leads to cell death by apoptosis [24, 25]. Tamoxifen is an antineoplastic agent that has affinity for estrogen receptors in mammary cells, thus turning to be an antagonist of cell growth. Doxorubicin acts on three different fronts (i) by altering the fluidity of the cell membrane; (ii) by forming free radicals by enzymatic reduction; and finally (iii) by blocking the DNA and RNA synthesis through the reduction the topoisomerase II activity [24, 26]. Cisplatin’s mechanism is related to the selective inhibition of DNA synthesis because of its alkylating activity [27]. 5-fluorouracyl is considered to be a drug belonging to the antimetabolite class capable of chemically blocking DNA synthesis as it is a base analogue. However, for its activity to occur, 5-fluorouracyl must be converted into its metabolites and these, in turn, incorporated into RNA (fluorouridine triphosphate - FUTP) or DNA (fluorouridine deoxyuridine triphosphate - FdUTP) in the presence of amino acid cysteine, thus preventing cell replication [7, 28].

Our results have demonstrated that 1 can induce cell death and improve the action of irinotecan, tamoxifen and doxorubicin by additive effect and/or synergism; and that 2 may favor the effects of tamoxifen and cisplatin by additive effect, and of irinotecan and doxorubicin by synergism. Regarding 5-fluouracyl, it was observed that the two sesquiterpene lactones are able to influence its mechanism of action and thus reduce its ability to cause cell death indicating antagonism in association with either 1 or 2. The same antagonistic effect occurred in the combination of cisplatin and 1.

Concerning the mechanism of action of sesquiterpene lactones, studies have demonstrate, in general, that they act in the signaling pathway of NF-kB, a transcription factor that regulates the expression of more than 150 genes related to the immune response, to intracellular stress, to stimulation of cell division and to inhibition of apoptosis (Heyden and Ghosh, 2008; Li, et al., 2015). It is inferred that due to the presence of the double bond conjugated with the carbonyl, compound 2 acts on the NF-kB signaling pathway through alkylation, via Michel addition, in the presence of α-methylene-γ-lactone rings. This alkylation leads to the covalent bond of the p65 subunit of NF-kB and cysteine, resulting in a conformational change of NF-κB that inhibits its activity [22, 29]. Compound 1 has no double bond conjugated to a carbonyl in its structure, yet it negatively regulates an expression of TNF-α, cytokine is also regulated by the transcription factor NF-kB [13].

The cisplatin, that acts by selective inhibition of DNA synthesis owing to its alkylating activity [27], presented distinct results for 1 and 2, being an antagonist for the former and additive agent for the latter. It is therefore assumed that the alkylating activity can be influenced by sesquiterpene lactones. The antagonistic action of 1 and cisplatin allows us to infer that these two compounds compete for actions sites, which would reduce the cisplatin ability to cause cell death in the MCF-7 lineage. The same effect, however, was not observed in 2, a fact that could be related to the different alkylation sites of 1 and 2.

5-Fluorouracyl, that requires cysteine [28] to play an anti-cancer role, is believed to lose part of its activity because of the competition of the compound 2 for this amino acid. Thus, the more α-methylene-γ-lactone rings, the more alkylation events; a larger number of NF-κB enzymes is inhibited and a larger number of cysteines is required [22]. Following this logic, it is inferred that there is reduced intracellular cysteine bioavailability to make the mechanisms of action of 5-fluorouracyl effective in the presence of 2, which explains the antagonistic effects observed in this study.

The 5-fluorouracyl has a mechanism of action very similar to the compounds tested, suppressing the transcription factor NF-kB. The antagonistic action of the association between 5-fluorouracyl and compound 1 is present in the lowest doses (40; 100 and 200 μM); but, in the highest concentrations (300 and 400 μM) a synergistic action is observed. This synergism is explained by the combined action of the tested compounds on the reduction of the expression of genes involved in cell proliferation [14]. However, the antagonism of the lower doses should be better studied with different concentrations and in other cell lines.

Even with differentiated responses, the combination of sesquiterpene lactones and doxorubicin can lead to certain linearity, that is, the associations showed synergistic activity with 1 and 2. This fact determined the choice of this anticancer agent for the assays of evaluation of death and cell proliferation.

In this assay three concentrations of 1 and 2, within the IC_50_ range, were tested in combination with doxorubicin in two experimental protocols: (i) continuous treatment for 4 h and (ii) continuous treatment for 4 h followed by a 24 h-post-incubation in drug-free medium. In carrying out the protocol, it was investigated whether, by removing the treatment, the effects are reduced by DNA repair or if they are potentiated, since the primary lesions may become secondary as a result of an attempted repair [22].

In both experiments, 2 presented higher apoptotic activity, and in the post-incubation treatments an increase occurred in cell death when compared with the 4 h-protocol. Differently, after the post-incubation period, 1 did not cause an enhanced reduction in cell viability as 2 did. These events can be elucidated if the structures of 1 and 2 are taken into account. The sesquiterpene lactone 1 has no exocyclic double bond, which could explain the different mechanisms of action presented. This fact is corroborated by the higher cytotoxic efficacy of 2 in previous studies [10].

In this same assay, when there is a combination of lactones with doxorubicin, an increase in the average number of cells in apoptosis is observed. However there is no dose-dependent response in either of the two evaluated compounds. When the capacity of damage potentiation after 24 h of treatment is evaluated, the phenomenon is seen to occur only in the lowest doses of the compounds when tested alone, and in the highest doses when tested in association with the chemotherapeutic.

These events are believed to be closely related to the different mechanisms of action of the tested chemotherapeutic agents and compounds, corroborating the hypothesis that, because they act in different metabolic pathways of death and mechanisms of cell cycle arrest, they are complementary and can favor the development of new anticancer therapies.

In addition, both 1 and 2 have effective antiproliferative action. In combined treatments, a reduced cell proliferation was observed for both isolates. Although 2 showed more successful results when isolated or associated with doxorubicin. These events corroborate the results obtained in the previous assays, indicating that compounds 1 and 2 act as antiproliferative agents by activating apoptotic events.

## Conclusions

Finally, it can be deduced that sesquiterpene lactones have therapeutic potential for developing anticancer therapies. It is also noteworthy that these compounds can be used in combination with doxorubicin, since they are capable of causing changes in DNA synthesis and regulating the apoptosis mechanism, events that, through the action of the sesquiterpene lactones, lead to reduced toxicity.

Furthermore, the tested compounds have the ability to exert a synergistic action on doxorubicin, possibly by down-regulating the drug resistance mechanisms. However, it is observed that compound 2 is highlighted in its action because smaller doses are required for a performance with good efficiency, unlike compound 1. All the points aforementioned lead to the conclusion that the studied sesquiterpene lactones, especially isolated compound 2, have therapeutic potential for isolated use and/or combined application with irinotecan, tamoxifen and, especially, doxorubicin.

## Abbreviations

1: 11 13 – diidrozaluzanin C
2: Gocnatiolide C
Brdu: 5 – bromodeoxyuridine
CI: Combination Index
FA: Affected cell fraction
h: Hours
MARR: Mitotic Activity Reduction Ratio
MTT: 3-(4,5-dimetilltiazol-2-il)-2,5-difeniltetrazol bromide
